# Interaction of DNA with Simple and Mixed Ligand Copper(II) Complexes of 1,10-Phenanthrolines as Studied by DNA-Fiber EPR Spectroscopy

**DOI:** 10.3390/ijms160922754

**Published:** 2015-09-21

**Authors:** Makoto Chikira, Chew Hee Ng, Mallayan Palaniandavar

**Affiliations:** 1 Department of Applied Chemistry, Chuo University, Kasuga, Bunkyou-ku, Tokyo 112-8551, Japan; 2 Department of Pharmaceutical Chemistry, International Medical University, Kuala Lumpur 57000, Malaysia; E-Mail: NgChewHee@imu.edu.my; 3 Department of Chemistry, Indian Institute of Technology Bombay, Powai, Mumbai 400 076, India; E-Mail: palaniandavarm@gmail.com

**Keywords:** electron paramagnetic resonance, DNA fiber, Cu(II) complexes, 1,10-phenanthrolines, DNA binding structures, DNA conformation

## Abstract

The interaction of simple and ternary Cu(II) complexes of 1,10-phenanthrolines with DNA has been studied extensively because of their various interesting and important functions such as DNA cleavage activity, cytotoxicity towards cancer cells, and DNA based asymmetric catalysis. Such functions are closely related to the DNA binding modes of the complexes such as intercalation, groove binding, and electrostatic surface binding. A variety of spectroscopic methods have been used to study the DNA binding mode of the Cu(II) complexes. Of all these methods, DNA-fiber electron paramagnetic resonance (EPR) spectroscopy affords unique information on the DNA binding structures of the complexes. In this review we summarize the results of our DNA-fiber EPR studies on the DNA binding structure of the complexes and discuss them together with the data accumulated by using other measurements.

## 1. Introduction

The interaction and reaction of metal complexes with DNA have long been the subject of extensive investigation in relation to the development of new reagents for biotechnology and medicine [1,2]. Also, DNA–metal complex systems are very useful as asymmetric catalysts in organic synthesis [3,4,5,6]. Among the metal complexes investigated so far, Cu(II) complexes of 1,10-phenanthroline (phen) and their derivatives have attracted much attention as they function as chemical nucleases. Sigman *et al.* demonstrated that [Cu(phen)_2_]^+^ complex inhibits DNA or RNA polymerase activities and induces strand scission of DNA in the presence of H_2_O_2_ or thiol [7,8] by catalyzing the formation of reactive oxygen species (ROS), which involves Cu(II)/Cu(I) redox cycle [7,8,9,10,11,12,13]. Since then, various ternary complexes of Cu(II) and phen and its derivatives with other ligands have been studied and they are found to exhibit additional properties like antitumor, antiviral, and photochemical DNA cleavage activities[14,15,16,17,18,19,20,21,22,23].

To improve the functions of metal complexes for application in the field of biotechnology, medicine and organic synthesis, one has to tune their properties such as redox potential to activate or deactivate oxidants, quantum yield in photochemical reactions, p*K*_a_ of ligands in hydrolytic cleavage of nucleic acids, and hydrogen-bonding network in recognizing specific nucleotide base sequences, and their availability in drug delivery system. Also, one should study how do the DNA binding structures of complexes correlate to their reactions with DNA. For example, the coordination geometry of a Cu(II) complex bound to DNA affects the Cu(II)/Cu(I) redox behavior and a change in the coordination geometry has been found to primarily determine the properties of Cu(II) rather than Cu(I) complex species [24]. Although structures of DNA-bound Cu(II) complexes of phen and phen derivatives have been proposed, the geometrical factors that determine the binding mode of the complexes are seldom reported.

Among the various spectroscopic methods used to assess the DNA bound structures of paramagnetic metal complexes, DNA-fiber electron paramagnetic resonance (EPR) spectroscopy affords unique information on the binding structures, stereo-specificity, and dynamic properties of the complexes bound to DNA [25,26,27,28]. By studying the changes in EPR line shapes upon varying the orientation of the DNA fibers in a magnetic field, one can estimate the orientations of the principal axes of the magnetic tensors, such as *g*, *A* and *D*, relative to the DNA double-helical axis. The *g* and *A* tensors reflect the strength and symmetry of the ligand field while the *D* tensor determines the magnitude of anisotropic spin–spin interaction if the complex has spin quantum number higher than 1/2 [29]. In addition, an analysis of the temperature-dependent EPR line shapes would provide information on the motion of the complexes on DNA [30].

In this review, we summarize the results of our DNA-fiber EPR studies on the DNA-bound structures of 1:1 complexes of Cu(II) with phen and its methyl derivatives, ternary complexes with amino acids, [Cu(phen)(AA)]^+^, Cu(II) complexes of phen-derived alkyl amine, [Cu(phen)_2_(H_2_O)]^2+^, ternary Cu(II)-phen complexes with ethylenediaminediacetic acid (edda), [Cu(phen)(edda)], and ternary Cu(II)-phen complexes with cationic Schiff base ligands. To assess the importance of the fused aromatic rings of phen in determining the DNA binding mode of [Cu(phen)(H_2_O)_3_]^2+^, the binding structure of the analogous complex [Cu(bpy)(H_2_O)_3_]^2+^, where bpy is 2,2′-dipyridine, is also described.

## 2. DNA-Fiber EPR Spectra of Cu(II) Complexes

**Figure 1:**
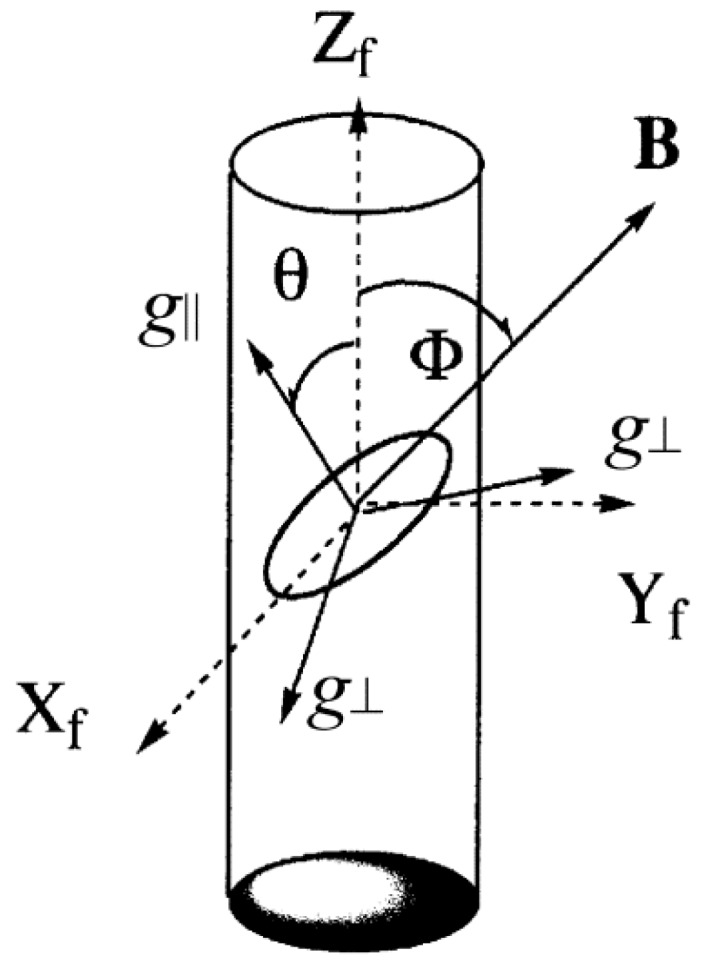
Coordinate systems: **B**, static magnetic field; (X_f_, Y_f_, Z_f_), DNA-fiber axes; (*g*_||_, *g*⊥), *g* tensor axes; Ф, the angle between **B** and Z_f_; θ, the angle between *g*_||_ axis and *Z*_f_.

If a Cu(II) complex binds with a specific orientation to double-helical DNA, as schematically shown in Figure 1, and the double helices are highly oriented in the DNA fibers, the EPR spectrum will change as a function of the angle Ф between the DNA fiber axis (*Z*_f_) and the static magnetic field (**B**). Simulations of the experimental spectra obtained from such studies can provide information on the orientation and randomness of the complexes bound to double-helical DNA. The orientation of a complex on the double-helical DNA is specified by an average angle θ_0_ and the randomness of θ by Δθ on the assumption that the distribution of θ is represented by a Gaussian function, as expressed by Equation (1):

(1) $$G\left(\text{θ}\right)=\frac{1}{\text{Δθ}}\text{exp}\left(\frac{-{\left(\text{θ}-{\text{θ}}_{0}\right)}^{2}}{2{\left(\text{Δθ}\right)}^{2}}\right)$$

**Figure 2:**
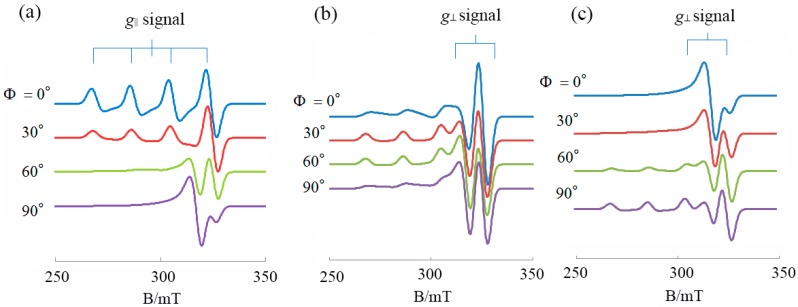
DNA fiber EPR spectra calculated for a planar Cu(II) complex. (**a**) θ_0_ = 0°; (**b**) θ_0_ = 45°; (**c**) θ_0_ = 90°; Δθ = 20°; *g*_||_ = 2.20, *g*⊥  = 2.05; *A*_||_ =180.0 G, $A_{⊥}$ = 10.0 G, $A_{N||}$ = 10.0 G, $A_{N⊥}$ = 10.0 G; Δ*B*_||_ = 25.0 G, Δ$B_{⊥}$ = 30.0 G; Microwave frequency = 9.1 GHz. (Δ*B*_||_ and Δ$B_{⊥}$ are anisotropic line widths for the *g*_||_ and *g*⊥ directions, respectively).

Typical DNA-fiber EPR spectra calculated for a planar Cu(II) complex with the *g*_||_ axis perpendicular to the coordination plane are shown in Figure 2. The details of the calculations have been reported elsewhere [31]. As shown in Figure 2a, when the *g*_||_ axis is parallel to *Z*_f_ axis (θ_0_ = 0°), the intensity of the *g*_||_ signal has its maximum at Ф = 0° and minimum at Ф = 90°, and *vice versa* for the *g*⊥  signal. On the other hand, when the *g*_||_ axis is perpendicular to *Z*_f_ axis (θ_0_ = 90°, Figure 2c), the intensity of the *g*⊥ signal has its maximum at Ф = 0° and minimum at Ф = 90°, and *vice versa* for the *g*_||_ signal. When the *g*_||_ axis is inclined by 45° from *Z*_f_ axis (Figure 2b), the intensity of the *g*_||_ signal has its maximum when Ф = 30°–60°.

If the complexes are fixed randomly on the DNA double helix, the spectrum becomes independent of Ф and the line shapes show the same patterns as those of frozen solutions or powders. If the complexes are mobile on DNA relative to the EPR time scale, EPR spectra are observed with line shapes the same as those of the solutions [30]. Both the stereo-specific binding and the dynamic motion of the complex are important in regulating the interaction or reaction of metal complexes with DNA.

Also, it has been shown that the conformation of double-helical DNA changes with the humidity around DNA fibers. DNA assumes B-conformation at a relative humidity higher than 92% but A-conformation below 75% [32]. The orientation of Cu(II) complexes bound to DNA changes with such conformational changes. Details of the preparation of DNA fibers containing copper complexes at different humidities and the EPR measurements have been described elsewhere [31,33].

## 3. Interaction of Cu(II) Complexes of Phen and Its Derivatives with DNA

### 3.1. 1:1 Complexes of Cu(II) with Phen and Its Methyl Derivatives

**Figure 3:**
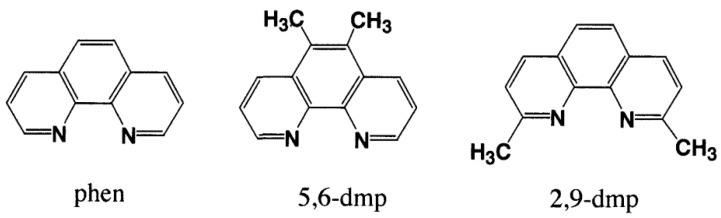
1,10-phenanthroline (phen) and its methyl derivatives.

To understand the role of phen and its derivatives on the interaction of Cu(II) complexes with DNA, the binding structures of 1:1 complexes of Cu(II) with phen and its methyl derivatives (Figure 3) have been studied.

These Cu(II) complexes in frozen aqueous solution show spectra with *g*_||_ > *g*⊥ corresponding to the presence of one unpaired electron in the *d_x_*^2^_− *y*_^2^ orbital and the coordination geometry around Cu(II) becomes square planar with tetrahedral or tetragonal-pyramidal distortion [34,35]. In an aqueous solution, the *X*^−^ anions coordinated in the solid state in [Cu(phen)(H_2_O)*X*_2_] are replaced with water, resulting in cationic aquated species that are likely to bind to the poly-anionic DNA. The change in the EPR spectra with pH is attributed to the deprotonation of coordinated water molecules. In neutral and weak alkaline aqueous solutions, the formation of di-µ-hydroxo-bridged dinuclear complexes has been suggested [36,37]. Such dinuclear complexes have been isolated and characterized by single crystal X-ray diffraction technique [38], and it has been found that a water molecule or chloride anion is coordinated in the apical position of the two tetragonal Cu(II) centers to form tetragonal pyramidal structures. So it is suggested that a similar tetragonal pyramidal [Cu(phen)(H_2_O)_3_]^2+^ is formed in an aqueous solution. To confirm how favorable the tetragonal pyramidal structure is, the energy of the optimized structure of [Cu(phen)(H_2_O)_3_]^2+^ has been estimated by Density Functional Theory (DFT) calculation using Gaussian 09 Revision D.01 [39]. The estimated energy was compared with the energy of structure-optimized [Cu(phen)(H_2_O)_2_]^2+^ and a water molecule located in the axial position at 4 Å from the Cu(II) center of square planar [Cu(phen)(H_2_O)_2_]^2+^ (Figure S1a). The calculation converged to the tetragonal pyramidal structure (Figure S1b) with a stabilization energy of 170.8 kJ/mol.

A similar calculation for the optimized structure of [Cu(phen)(H_2_O)_3_]^2+^ and a water molecule (Figure S2a) converged to octahedral [Cu(phen)(H_2_O)_4_]^2+^ (Figure S2b with a stabilization energy of 48.1 kJ/mol). As shown in Figure S2b, the Cu–O bond length (2.37 Å) of one of the apically coordinated water molecules is much longer than the other (2.29 Å). Taking into account the negative entropy effect of water coordination, one can reasonably conclude that the octahedral form is not so stable as to maintain its structure when it binds to DNA. Therefore, the DNA binding of the tetragonal [Cu(phen)(H_2_O)_3_]^2+^ is focused.

**Figure 4:**
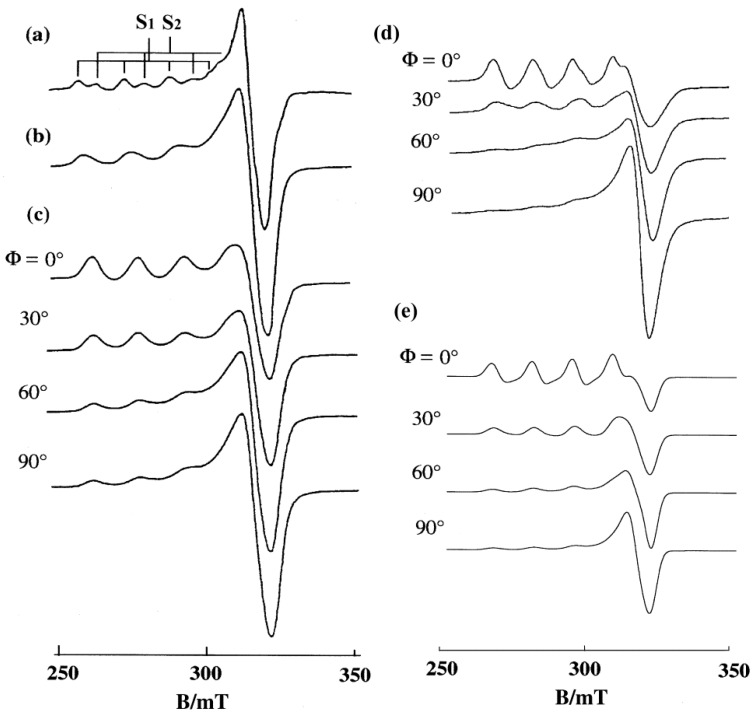
EPR spectra of [Cu(phen)(H_2_O)_3_]^2+^ (**a**) in frozen solution (20 mM, pH 7.0) at −150 °C, **S_1_**: *g*_||_ = 2.317, *A*_||_ = 0.0167 cm^−1^, **S_2_**: *g*_||_ = 2.260, *A*_||_ = 0.0172 cm^−1^; (**b**) in DNA pellet at −150 °C; and (**c**) on A-form DNA fibers at room temperature, [DNAbp]/[Cu(II)] = 25; (**d**) observed and (**e**) calculated EPR spectra of [Cu(phen)(H_2_O)_3_]^2+^ on B-form DNA fibers at room temperature. [DNA-bp]/[Cu(II)] = 25, [**G**]/[**I**] = 2. Other parameters used for the simulation: species **I**: θ_0_ = 6°, Δθ = 12°, Δ*B*_||_ *=* 40 G, Δ$B_{⊥}$ = 30 G, species **G**: θ_0_ = 30°, Δθ = 30°, Δ*B*_||_ *=* 40, Δ$B_{⊥}$ = 40 G, [**G**]/[**I**] = 2. For both **I** and **G**, *g*_||_ *=* 2.290, *g* ⊥  = 2.08, *A*_||_ = 0.0154 cm^−1^, $A_{⊥}$ = 0.0010 cm^−1^, *A_N_*_||_ *=* 0.0010 cm^−1^, $A_{N⊥}$ = 0.0010 cm^−1^ [27].

The EPR spectra of mono-phen Cu(II) complex obtained in frozen solution, in DNA pellet, and on A-form DNA-fibers at room temperature are shown in Figure 4a–c [27]. The intensity of the *g*_||_ signal has its maximum at Ф = 0° and minimum at Ф = 90°, and *vice versa* for the *g*⊥ signal. When the DNA fibers are changed from A to B form, the line shape of the *g*_||_ signals at Ф = 0° appear similar to those observed for the complex diluted in a single crystal of the analogous diamagnetic complex and the intensity of the *g*_||_ signals decreases with Ф more conspicuously (Figure 4d). This indicates that the degree of orientation of the coordination plane considerably increases upon changing the DNA from A to B form, and the *g*_||_ axis is almost parallel to the DNA fiber axis. However, the intense signal of the fibers at Ф = 0° observed around 320 mT indicates that another species is bound on the DNA with the *g*_||_ axis inclined considerably from the DNA fiber axis. The observed spectra were simulated with θ_0_ = 6° and Δθ = 12° for one species (**I**), and with θ_0_ = 40° and Δθ = 30° for the other species (**G**), assuming that the ratio of the amount of species [**G**]/[**I**] = 2 and using the same *g* and *A* values for both **I** and **G** (Figure 4e). As the calculated spectra are not affected in shape by Δθ values larger than 30° for **G**, the uniqueness of the orientation of **G** could not be assessed quantitatively from the EPR spectra.

The ratio of the amount of the two differently oriented species [**G**]/[**I**] changes upon varying the substituents on the phen ring. In the case of mono 5,6-dmp Cu(II) complex, where 5,6-dmp is 5,6-dimethyl-1,10-phenanthroline, the *g*⊥ signal around 320 mT at Ф = 0° or the *g*_||_ signal at Ф = 90° decreases in intensity considerably as compared to those of the monophen Cu(II) complex (Figure S3).

The spectra calculated by assuming [**G**]/[**I**] = 1.5 fit well with the observed spectra with the same parameters used for mono-phen Cu(II) complex. These results clearly indicate that the methyl groups at 5,6-positions of phen ring contribute to the increase in the amount of species **I** whose *g*_||_ axis is almost parallel to the fiber axis. As the *g*_||_ axis is assigned to a molecular axis perpendicular to the Cu(II) coordination plane, which is almost parallel to the phen plane, one could reasonably conclude that the phen plane is oriented parallel to the base-pair plane. The most probable binding structure is the one in which the phen plane is intercalated between the base pairs of double-helical DNA. It should be noted, however, that mono-phen or mono-5,6-dmp Cu(II) complex binds also non-intercalatively in large amounts with the phen plane tilted with respect to the base-pair planes (species **G**).

To understand these results in more detail, the binding of the [Cu(phen)](H_2_O)_3_]^2+^ and [Cu(5,6-dmp)(H_2_O)_3_]^2+^ complexes to a double stranded oligonucleotide 5′-dCGCGAATTCGCG (odn1) was studied by molecular dynamic calculations using AMBER14 [40], Gaussian09 [39] and RESP-ESP charge Derive Server [41]. The details of the calculation are described in the Supplementary Materials. The preliminary results suggest that a stable intercalated structure corresponding to the species **I** exists for [Cu(phen)(OH_2_)_3_]^2+^ (Figure S4a). The structure was obtained by inserting the phen plane manually between the central AT base pairs of odn1 from the minor groove side by using xleap tool in Amber14. It should be noted that the apically coordinated water molecule stays in the groove and does not interfere with the intercalative binding of the phen moiety. If the calculation is made by simply positioning the phen plane in the minor groove of the double stranded odn1, the same energy minimization and molecular dynamic calculations lead to a structure with the phen plane oriented along the minor groove (Figure S4b), suggesting that the minor groove bound species is one of the components of species **G** observed in the DNA fiber EPR.

The intercalated structure of [Cu(5,6-dmp)(H_2_O)_3_]^2+^ has been also obtained (Figure S4c), which indicates that the methyl groups on 5,6 positions of phen penetrate well into the major groove side without any particular steric hindrance from the neighboring nucleobase or ribose moieties. It is revealed that the complex [Cu(5,6-dmp)_2_(H_2_O)]^2+^ induces a conformational transition of DNA from B to Z form, where the methyl groups on one phen ring are assumed to prevent the other phen ring from being involved in intercalative interaction [42]. The present results indicate that the methyl groups on the 5,6 positions of phen do not interfere with the intercalative binding from the minor groove as far as the [Cu(5,6-dmp)(H_2_O)_3_]^2+^ complex is concerned.

**Figure 5:**
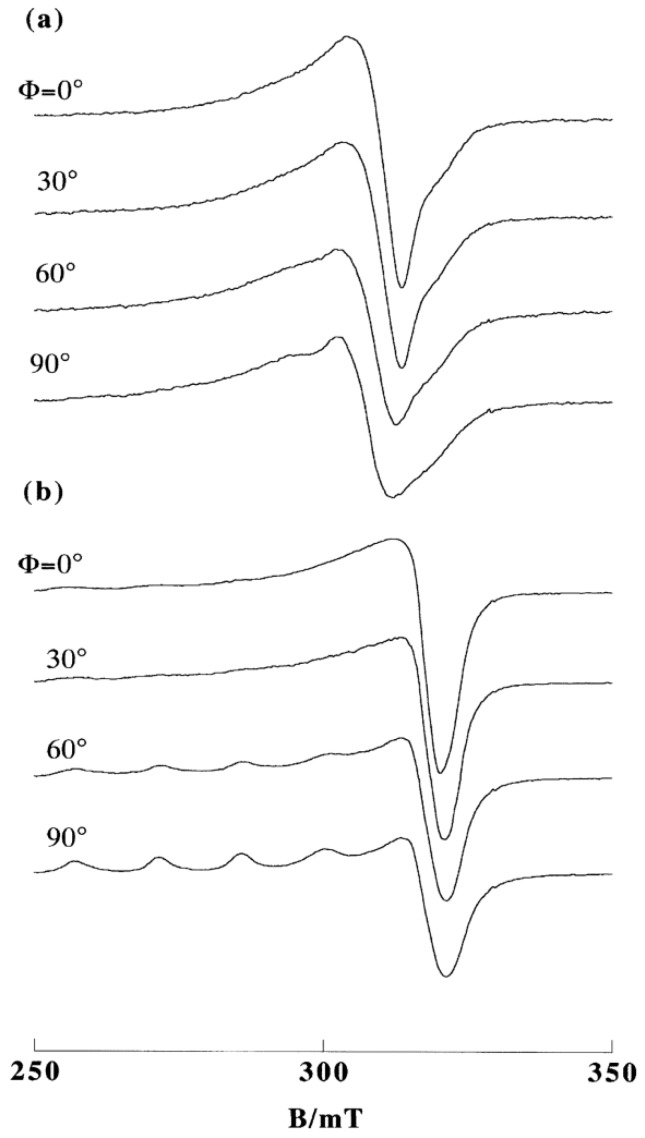
EPR spectra of [Cu(2,9-dmp)(H_2_O)_3_]^2+^ on B-form DNA fibers at (**a**) room temperature and (**b**) −150 °C. [DNA-bp]/[Cu(II)] = 30 [27].

In contrast to the methyl groups at the 5,6 positions of phen in [Cu(2,9-dmp)(H_2_O)_3_]^2+^, those at the 2,9-positions in [Cu(2,9-dmp)(H_2_O)_3_]^2+^ cause a dramatic change in the EPR line shapes. The intensities of *g*_||_ signals of [Cu(2,9-dmp)(H_2_O)_3_]^2+^ on A-form DNA-fibers become the smallest at Ф = θ° and the largest at Ф = 90° (Figure S5c). This indicates that there is a species whose *g*_||_ axis is almost perpendicular to the DNA-fiber axis. Also, a conformational change of the DNA fibers from A to B form causes changes in the spectral features at room temperature into single broad peaks without copper hyperfine splitting (Figure 5a). On freezing the B-form DNA fibers at −150 °C, the hyperfine splitting of the *g*_||_ signal emerges prominently at Ф = 90° (Figure 5b) and the intensity of the *g*_||_ signal becomes Ф-dependent again and decreases to a minimum at Ф = 0°.

**Figure 6:**
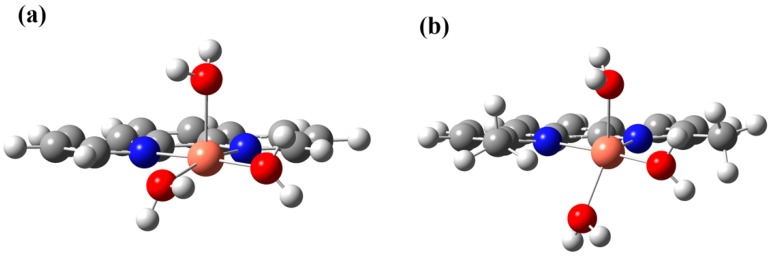
Optimized structure of (**a**) [Cu(phen)(H_2_O)_3_]^2+^ and (**b**) [Cu(2,9-dmp)(H_2_O)_3_]^2+^. The color of the atoms: orange, Cu; blue, N; red, O; gray, C; white, H.

Preston *et al.* have reported that [Cu(2,9-dmp)(H_2_O)Cl_2_] with a [CuN_2_OCl_2_] chromophore has a trigonal bipyramidal geometry in the solid state, in which one of the Cu–N distances of 2,9-dmp is longer (2.24 Å) than the other (1.98 Å) [43]. To confirm if [Cu(2,9-dmp)(H_2_O)_3_]^2+^ has a similar trigonal bipyramidal geometry, DFT calculations were undertaken by using Gaussian09 [39] and the results are shown together with the calculated structure of [Cu(phen)(H_2_O)_3_]^2+^ in Figure 6. The τ values [44] of [Cu(phen)(H_2_O)_3_]^2+^ and [Cu(2,9-dmp)(H_2_O)_3_]^2+^ are 0.182 and 0.751, respectively, indicating that the structure of [Cu(phen)(H_2_O)_3_]^2+^ is best described as a trigonal bipyramidal distorted square-based pyramidal (TBDSBP) while that of [Cu(2,9-dmp)(H_2_O)_3_]^2+^ as square pyramidal distorted trigonal bipyramidal (SPDTB). As in the case of [Cu(2,9-dmp)(H_2_O)Cl_2_], one of the Cu–N distances (2.08 Å) in [Cu(2,9-dmp)(H_2_O)_3_]^2+^ is longer than the other (1.96 Å).

The EPR spectrum of [Cu(2,9-dmp)(H_2_O)_3_]^2+^ in the DNA pellet at low temperature (Figure S5b) shows an axially symmetric line shape with the spectral parameters (*g*_||_ = 2.346, *A*_||_ = 0.0140 cm^−1^), indicative of a square pyramidal coordination. However, the broad spectra observed at room temperature for the B-form DNA fibers (Figure 5a) indicate that the complex rotates on the DNA or fluctuates between TBDSBP and SPDTB forms in such an EPR time scale that averages the anisotropy the *g* and *A*-tensors [30]. The B-form DNA fibers contain much water and behave like liquid crystals, in which a weakly bound complex has considerable freedom of motion. The ^31^P-NMR spectra of various DNA fibers reveal that the DNA double-helical structures have an inhomogeneity determined by base sequence and there remains the freedom of rotational motion around the phosphodiester moiety in the B-form DNA [32]. The presence of the mobile species inferred from EPR spectra may reflect such dynamic motions of the nucleotide phosphate ribose backbones.

The intercalated form of [Cu(2,9-dmp)(H_2_O)_3_]^2+^ shown in Figure S6a is obtained by MD/QM calculation starting from the initial structure constructed as in the case of [Cu(phen)(H_2_O)_3_]^2+^. However, when the phen plane of [Cu(2,9-dmp)(H_2_O)_3_]^2+^ is simply located in the minor groove at the starting point of the MD calculation, the complex came out of the groove after about 22 ns of MD calculation, as shown in Figure S6b.

The observed Ф-dependence of the EPR spectra of mono-2,9-dmp Cu(II) complex on A-form DNA fibers (Figure S5c) at room temperature and on B-form DNA fibers at −150 °C (Figure 5b) can be interpreted by the models, where phen plane intercalates to DNA and the longer Cu–N bond axis, which can be assigned as the *g*_||_ axis, is oriented perpendicular to the DNA-fiber axis. The τ values (Figure S7) estimated from the QM/MD calculation for the intercalated species change as shown in Figure S8 with the average value being 0.986, indicating that the trigonal pyramidal form is maintained during this period. Considering the results of MD calculation for the groove-bound species, the dynamic behavior of [Cu(2,9-dmp)(H_2_O)_3_]^2+^ in B-form DNA fibers at room temperature may correspond to the species whose phen moiety comes out of the intercalated position and rotates in a cavity of B-form DNA fibers. Though the whole process of intercalation and groove binding could not be reproduced by MM/MD/QM calculations, these results provide a better view of what is going on in the DNA fibers.

In the above discussions, we have concentrated on the binding of [Cu(phen)(H_2_O)_3_]^2+^, [Cu(5,6-dmp)(H_2_O)_3_]^2+^, and [Cu(2,9-dmp)(H_2_O)_3_]^2+^ from a minor groove side of odn1. It will be interesting to investigate the following aspects further.

(1). It is not clear if mono-phen Cu(II) complex intercalates as [Cu(phen)(H_2_O)_2_]^2+^ after removing the apically coordinated water molecule from [Cu(phen)(H_2_O)_3_]^2+^ or as [Cu(phen)(H_2_O)_4_]^2+^ keeping the two apically coordinated water molecules. At pH 7.0, the presence of [Cu(phen)(H_2_O)_2_(OH)]^+^, [Cu(5,6-dmp)(H_2_O)_2_(OH)]^+^, or [Cu(2,9-dmp)(H_2_O)_2_(OH)]^+^ should also be considered. The positive charge of the complexes decreases by dissociation of a proton from the coordinating water molecule, which does not favor binding to a negatively charged oligo-nucleotide. However, the removal of an apically coordinated water molecule results in the formation of flat coordination plane, which is favorable for intercalative binding.
(2). It would be interesting to investigate whether one of the coordinating water molecules in the complex is substituted with phosphate oxygen or N7 of the guanine residue to produce a covalent interaction.
(3). It is also important to investigate how the binding affinity changes when effecting changes in the base sequence of nucleotides.

In order to obtain answers for the questions above, one has to compare the thermodynamic free energy of binding for each binding mode; this is our aim for future investigation. These aspects should be also interesting to study for the complexes discussed in the subsequent sections.

### 3.2. Ternary Complexes with Amino Acids, [Cu(phen)(AA)]^n+^(n = 1 or 2)

The ternary Cu(II) complexes of 4,7-dmp and amino acids have been reported to exhibit cytotoxicity [23]. The ROS generation, which leads to initial genetic damage, might be the cause of cytotoxicity. Also, when cancer cells are incubated with [Cu(phen)(AA)(H_2_O)]^+^ (AA = methylated glycine: *viz*. sar, dmg, ala) both the generation of ROS and double-stranded DNA breaks occur in the cells [45].

**Figure 7:**
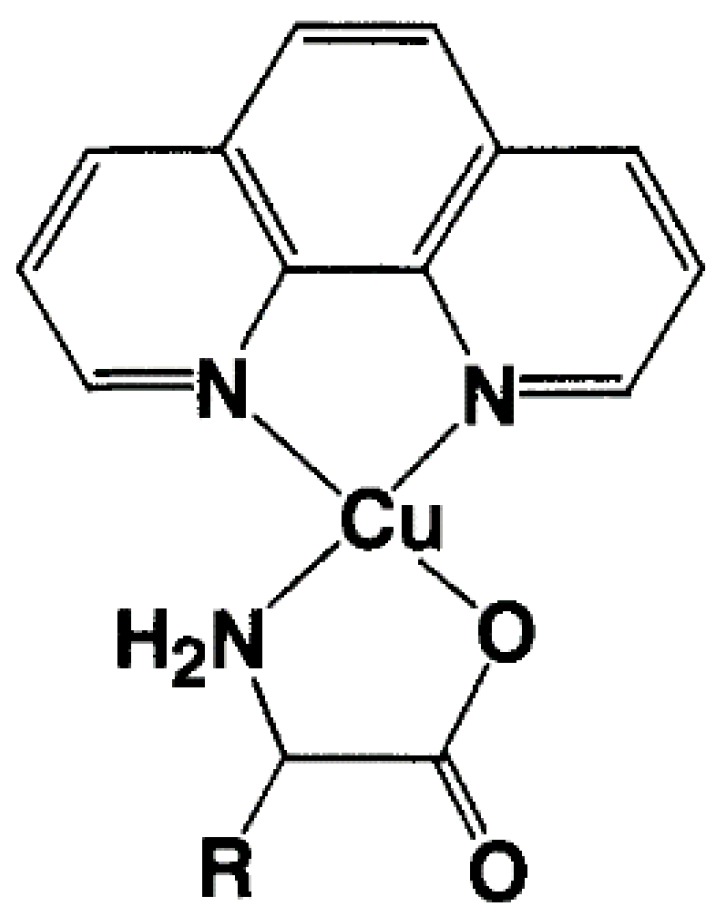
Ternary [Cu(phen)(AA)]^+^ complexes with amino acids (AA).

**Figure 8:**
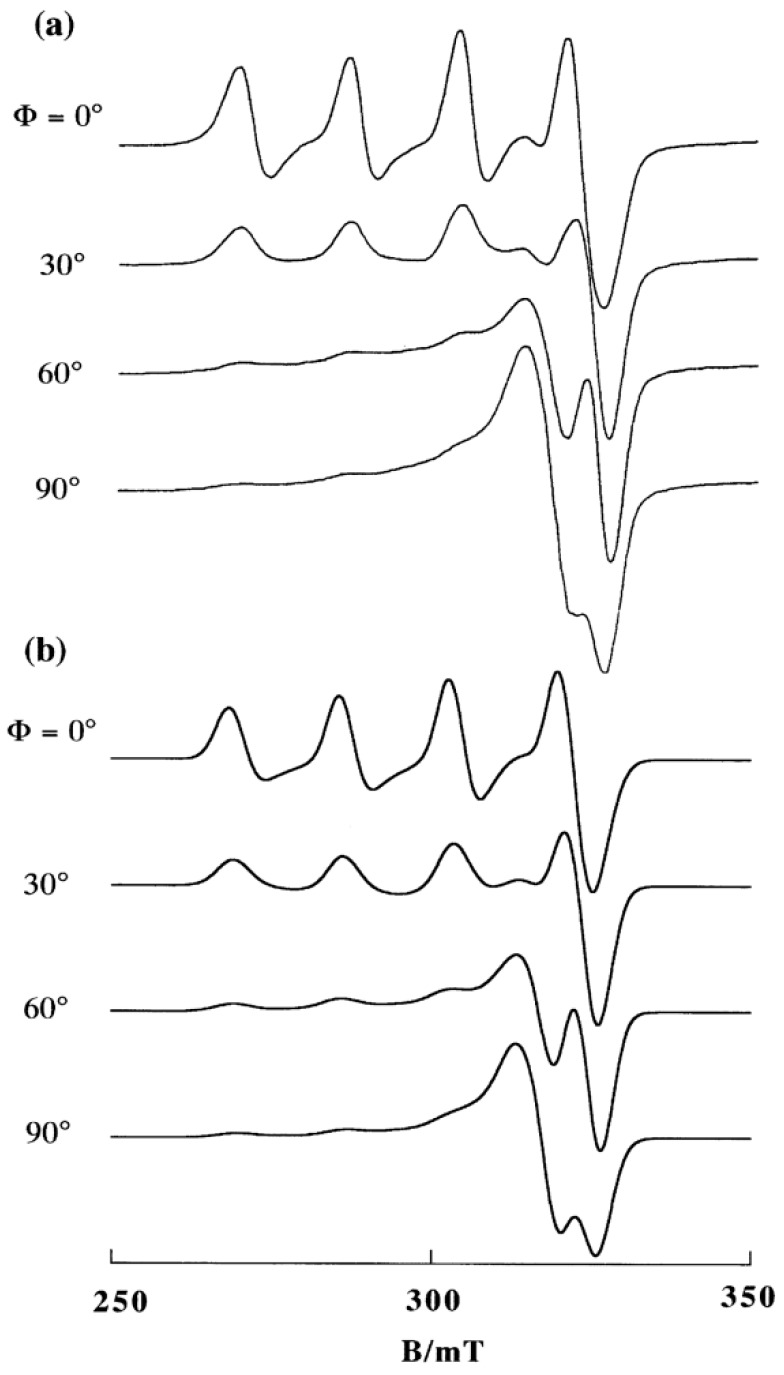
Observed (**a**) and calculated (**b**) EPR spectra of [Cu(phen)(Lys)]^2+^ on B-form DNA fibers at room temperature. [DNA-bp]/[Cu(II)] = 30, species **I**: *g*_||_ = 2.23, *g*⊥  = 2.07, *A*_||_ = 0.0177 cm^−1^, $A_{⊥}$ = 0.0015 cm^−1^, *A_N_*_||_ = 0.0010 cm^−1^, $A_{N⊥}$ = 0.0010 cm^−1^, θ = 3°, Δθ = 15°, Δ*B*_||_ = 25 G, Δ$B_{⊥}$ = 30 G. Species **G**: *g*_||_ = 2.235, *g* ⊥  = 2.07, *A*_||_ = 0.0172 cm^−1^, $A_{⊥}$ = 0.0013 cm^−1^, *A_N_*_||_ = 0.0010 cm^−1^, $A_{N⊥}$ = 0.0010 cm^−1^, θ = 30°, Δθ = 30°, Δ*B*_||_ = 45 G, Δ$B_{⊥}$ = 45 G. [**G**]/[**I**] = 2 [27].

The EPR spectra of the [Cu(phen)(AA)]^+^ complexes (Figure 7) bound to DNA reveal that some of the complexes undergo partial dissociation to give the mono-phen Cu(II) complex and the amino acid on the DNA [27]. The ratio of the amount of the intact ternary complex to the dissociated one on DNA changes with the kind of amino acids. The ternary complexes of l-lysine, l-arginine and l-glutamine scarcely dissociate on DNA and show EPR line shapes with an enhanced Ф-dependence even on A-form DNA fibers. As Figure 8 shows, the EPR line shape of [Cu(phen)(Lys)]^+^ becomes sharper on the B-form DNA fibers and the single-crystal-like *g*_||_ signals observed at Ф = 0° become considerably weak at Ф = 90°. However, it should be noted that not all the l-lysine complex species are intercalated into DNA. There are two species **I** and **G** oriented differently as in the case of [Cu(phen)(H_2_O)_3_]^2+^ and [Cu(5,6-dmp)(H_2_O)_3_]^2+^. The observed spectra were successfully simulated with θ_0_ = 3° and Δθ = 15° for **I**, and θ_0_ = 45° and Δθ = 45° for **G**, assuming that [**G**]/[**I**] = 2. Thus, about two thirds of [Cu(phen)(Lys)]^2+^ are bound, with the phen plane considerably tilted from the DNA double-helical axis.

In contrast, there are two kinds of *g*_||_ signals for the ternary complexes with glycine, l-leucine, l-serine, l-threonine, l-cysteine, l-methionine, and l-asparagine. This indicates that a considerable amount of the chelated amino acids in the ternary complexes is displaced upon binding to DNA. The ternary complex of l-asparagine does not remain intact on DNA as much as the ternary complex of l-glutamine**,** which has a side chain group longer by only one CH_2_ unit than that in l-asparagine.

For the ternary Cu(II) complexes of phen with amino acids, the observation of the species **I**, whose *g*_||_ axis is almost parallel to the DNA double-helical axis, supports the possibility of intercalative binding of the phen moiety. Another species with *g*_||_ axis titled from the fiber axis is also present. The labile water molecule coordinated at the apical position in the ternary Cu(II) complexes is substituted by a base or phosphate group of DNA to give rise to a Cu(II) species with the *g*_||_ axis inclined from the DNA-fiber axis.

The fact that a considerable amount of [Cu(phen)(Lys)]^2+^ or [Cu(phen)(Arg)]^2+^ is bound non-intercalatively indicates that there are several binding sites on the DNA where the free energy change of the binding becomes comparable to or larger than that of intercalative binding. For these complexes, the non-intercalative binding could not be attributed to substitution of the amino acid with DNA. Other than the apical coordination mentioned above, another possibility for the non-intercalative binding is the one where the phen moiety is in the minor groove of DNA with the phen plane aligned along the groove. However, a relatively large Δθ value estimated for [Cu(phen)(Lys)]^2+^ suggests that the minor groove binding might not be solely responsible for the non-intercalative binding.

Based on the stability constants reported so far, the amount of dissociated species of the Cu(II) complexes in solution in the pH range 7.0–7.4 is negligibly small [46,47]. It should be noted that the same pH range was employed for the preparation of DNA fibers. Thus, the displacement of an amino acid from the coordination sphere upon binding to DNA should be followed by coordination of a nucleobase nitrogen and/or a phosphate oxygen in DNA to copper. Though the stability constants of the ternary complexes change with the amino acid, no correlation is found between the amount of dissociated species and the stability constants.

### 3.3. Cu(II) Complexes of 1,10-Phenanthroline-derived Alkyl Amine

**Figure 9:**
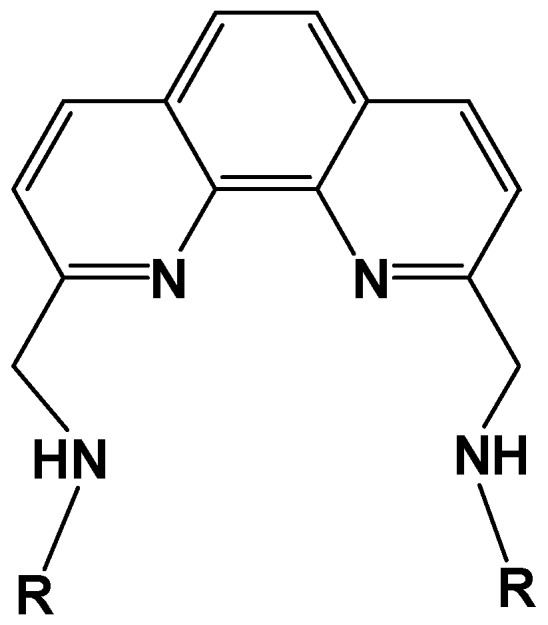
*N*,*N*′-dialkyl-1,10-phenanthroline-2,9-dimethanamine (L). *R* = methyl (**1**); *n*-propyl (**2**); isopropyl (**3**); *sec*-butyl (**4**); *tert*-butyl group (**5**). Numbers in the parentheses correspond to the respective Cu(II) complexes.

It has been shown that the methyl substituents on 2,9-positions of phen affects the structure of Cu(II) coordination sphere, resulting in a change in orientation of the *g* tensor axes on DNA [27]. These results prompted us to further investigate the effect of substituents at 2,9-positions. Wang *et al.* have prepared 1:1 Cu(II) complexes of *N*,*N*′-dialkyl-1,10-phenanthroline-2,9-dimethanamine (Figure 9) and studied the thermodynamic and kinetic properties of the complex–DNA binding [48]. They showed that the complexes interact with calf thymus DNA by both intercalative and covalent binding and that there are at least two steps in the binding process. In this section, we focus our attention on the structure of the complexes bound to DNA and the oxidative DNA cleavage effected with these complexes as well [28].

In the X-ray crystal structure of complex cation [Cu(LH)Cl_2_]^+^ (**2)** (LH is the protonated form of ligand L), Cu(II) is coordinated to two phen nitrogen atoms, one of the two secondary amine nitrogen atoms of the side chains, and two chloride ions. The coordination geometry of the Cu(II) is best described as trigonal bipyramidal distorted square based pyramidal (TBDSBP, Figure S9). Electronic and EPR spectral studies reveal that all the complexes in aqueous solution at pH~7.0 possess a CuN_3_O_2_ rather than CuN_4_O chromophore with one of the alkylamine side chains not involved in coordination. The structures of the complexes in aqueous solution at pH~7 change from distorted tetragonal to trigonal bipyramidal as the size of the alkyl group is increased.

In the A-form DNA-fibers, the complex **1** shows Ф-dependent EPR spectra in which the *g*_||_ signal becomes most intense at Ф = 0° (Figure S10). The conformational change of DNA-fiber from A to B form did not significantly affect the EPR line shape at either room or low (−150 °C) temperature (Figure S11). As the *g*_||_ axis is parallel to the normal of average coordination plane, one can reasonably conclude that the coordination plane of the species is oriented parallel to the DNA base pairs. These results indicate that a considerable amount of **1** is bound to DNA through the intercalative mode. The EPR spectra of **2** and **3** on B-form DNA-fibers are similar to those of **1**, indicating that the *n*-propyl or isopropyl groups do not interfere with the intercalative binding. Similar EPR spectra have been observed for the unsubstituted mono-phen Cu(II) complex and the ternary complexes, [Cu(phen)(AA)]*^n^*^+^, where H(AA) stands for amino acid [27]. The EPR spectra of **4** show line shapes a little different from those of **1**, **2** and **3**; an enhanced deformation of the complex toward trigonal bipyramidal geometry might have increased the rhombicity of the *g* tensor and/or the amount of non-intercalated species [28].

**Figure 10:**
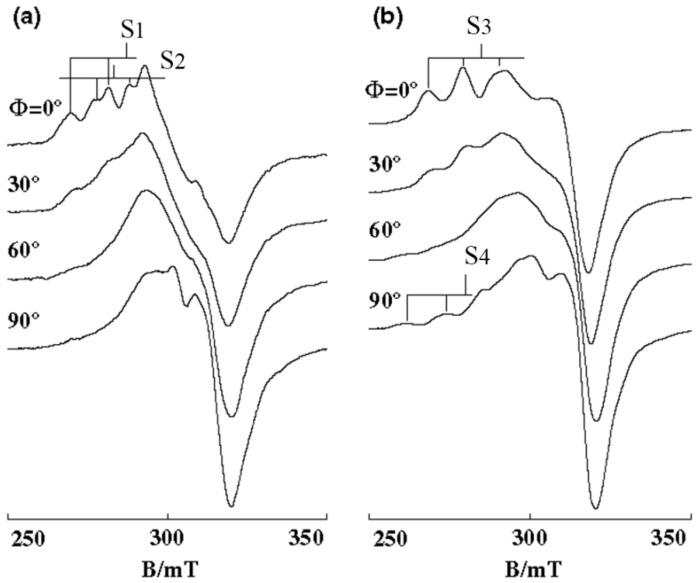
EPR spectra of **5** on B-form DNA-fibers at (**a**) room temperature and (**b**) −150 °C [28]. S3 is the overlapped *g*_||_ signal of S_1_ and S_2_.

The EPR spectra of **5**, with the bulkiest alkyl group among the present complexes, are quite different from those of **1**–**4**, which is consistent with the electronic spectral study. The complex **5** bound to B-form DNA fiber shows EPR line shapes with irregularly spaced hyperfine splitting (S_1_, S_2_) in the *g*_||_ region, indicating that several different species are bound with different *g* values and similar orientations on the DNA (Figure 10a). Freezing the B-form DNA fibers results in further changes in the EPR line shape; new *g*_||_ signals characteristic of the species with distorted tetragonal form emerge at lower magnetic field end of the spectra at Ф = 90° (Figure 10b, S_4_).

These results indicate that **5** is so flexible in coordination geometry that it could take various structures depending on both the temperature and DNA binding mode. It is obvious that such a structural change is caused by the interplay of DNA and the bulky *tert-*butyl groups, which interfere with the deep intercalation of the phen moiety only to lead the complex to move near to the surface of the double-helical DNA. As the orientation of *g* tensor of **5** on the molecular frame is not clear, it is difficult to describe the orientation of the phen plane of each species on the DNA. However, they are really bound stereospecifically on the DNA.

The observed changes in the physicochemical features of the complexes on binding to DNA suggest that all the complexes, except **5**, bind to DNA with partial intercalation of the derivatized phen ring in between the DNA base pairs. Electrochemical studies reveal that the complexes prefer to bind to DNA in the Cu(II) rather than the Cu(I) oxidation state [28]. Interestingly, **5** shows the highest DNA cleavage activity among all the present copper(II) complexes, suggesting that the bulky *N-tert*-butyl group plays an important role in modifying the coordination environment around the Cu(II) center, the Cu(II)/Cu(I) redox potential, and hence the formation of activated oxidant responsible for the DNA cleavage.

### 3.4. [Cu(phen)_2_(H_2_O)]^2+^

**Figure 11:**
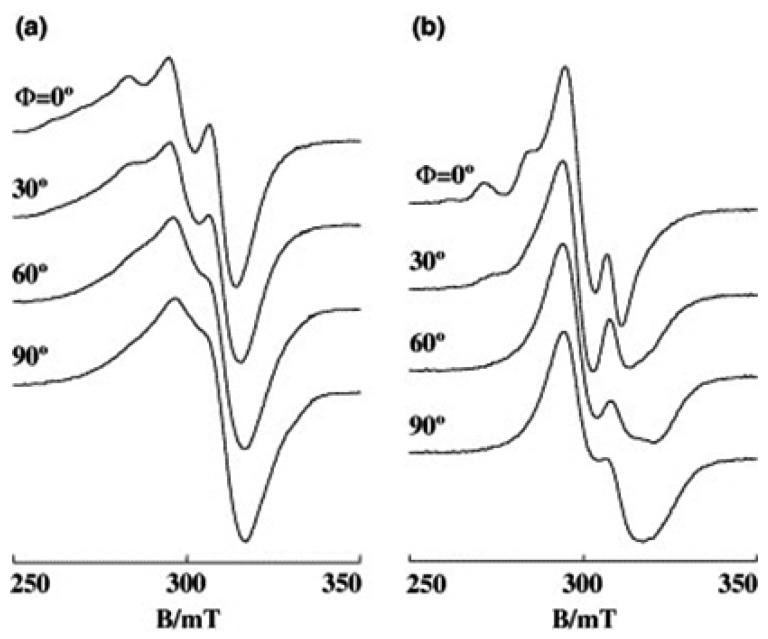
EPR spectra of [Cu(phen)_2_(H_2_O)]^2+^ on DNA fibers at room temperature: (**a**) A-form; (**b**) B-form [28].

The complex [Cu(phen)_2_(H_2_O)]^2+^ in frozen solution at pH 7.4 exhibits an EPR signal (*g*_||_ = 2.270) typical of a tetragonal pyramidal Cu(II) complex but the EPR line shapes are characteristic of the rhombic Cu(II) complex bound to DNA. The EPR spectra of the complex bound to A- and B-form DNA fibers change with Ф (Figure 11), indicating that the complex is stereospecifically orientated on the DNA. In addition, the conformational change of DNA from A to B form is accompanied by a structural change in the Cu(II) complex. The *g*_||_ signal at Ф = 0° disappears on freezing the B-form DNA fibers at −150 °C. It has been observed that crystallization of water in the B-form fibers at low temperatures sometimes disrupts the orientation of DNA and deforms the binding site, which accounts for the inhomogeneous broadening of the spectra [49]. These changes in the EPR spectra suggest that the coordination geometry of [Cu(phen)_2_(H_2_O)]^2+^ is flexible enough to tune the structure to suit the binding site of DNA. Actually, Hathaway and his collaborators have reported that structural distortions in [Cu(phen)_2_*X*][*Y*] complexes (*X* = Cl^−^, Br^−^, *Y* = various negative ions) depend upon *X* and *Y*, indicating that the copper coordination spheres are flexible and change with the environment [50,51]. It has been suggested that the cuprous complex [Cu(phen)_2_]^+^ cleaves B-form DNA much faster than it does A-form DNA because the complex has stronger affinity to the minor groove of B-form DNA than that of A-form DNA [52,53,54,55,56]. In addition, the difference in the coordination structures of [Cu(phen)_2_(H_2_O)]^2+^ bound to A- and B-forms of DNA might be another factor responsible for the difference in the rate of cleavage.

Robertazzi *et al.* calculated the binding structure of [Cu(phen)_2_(H_2_O)]^+^ bound to an oligonucleotide by steered molecular dynamics (SMD) simulations and concluded that one phen tightly fits inside the minor groove, guiding the copper center close to the atoms that undergo oxidative attack [57]. They have also concluded that the binding progresses exclusively in the minor groove and not in the major groove. In the oxidative DNA cleavage reactions with [Cu(phen)_2_(H_2_O)]^2+^, the complex is incubated first with DNA and then some reducing reagent is added to the solution to reduce the complex to [Cu(phen)_2_]^+^. The flexibility of [Cu(phen)_2_(H_2_O)]^2+^ bound to DNA will decrease the activation energy for the redox cycling between Cu(II) and Cu(I) species during DNA cleavage.

### 3.5. Ternary Copper(II)–Phen–Edda Complex [Cu(phen)(edda)]

A series of ternary metal(II) complexes of the type [M(phen)(edda)] (M = Cu(II), Co(III), Zn(II), Ni(II), phen is 1,10-phenanthroline, and edda = *N*,*N*′-ethylene-bridged diglycine or ethylenediamine-tetraacetic acid) have been synthesized and characterized by elemental analysis, FTIR, UV-visible spectroscopy, and magnetic susceptibility measurement [58]. The results of MTT assay [59] of these complexes on MCF-7 cancer cells reveal significant enhancement in their antiproliferative property, which results from synergy between the metal and ligands [25,58]. Preliminary results from apoptosis and cell cycle analysis with flow cytometry show that [Cu(phen)(edda)] partially induces cell cycle arrest at Go/G1 phase, which has been associated with activation of cell cycle checkpoint due to DNA damage [25].

**Figure 12:**
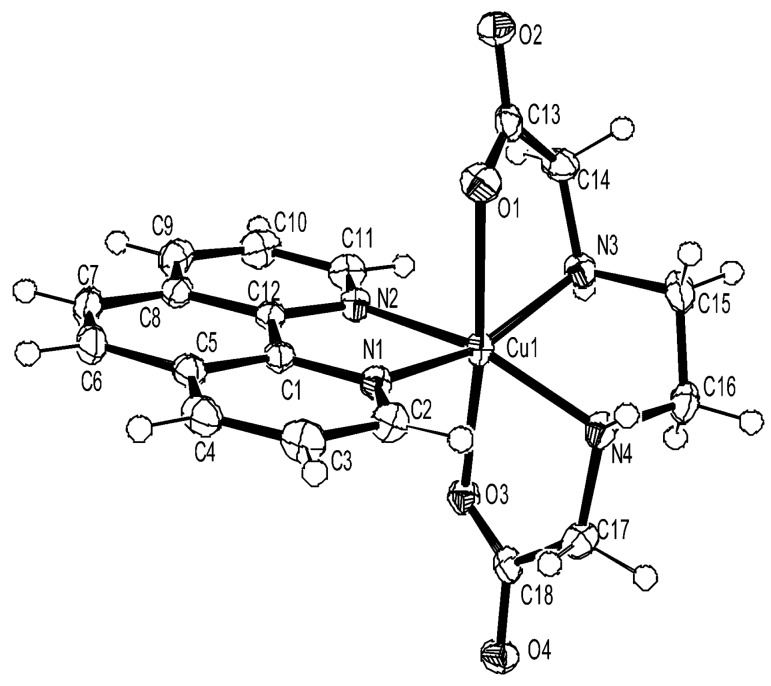
ORTEP view of ternary Copper(II)-phen-edda Complex [Cu(phen)(edda)] [25].

As shown in Figure 12, the X-ray crystal structure of [Cu(phen)(edda)] reveals the octahedral geometry around Cu(II). The coordinated carboxylate oxygen atoms O1 and O3 of the tetradentate edda ligand are *trans* to each other, with a O1–Cu1–O3 bond angle of 174.02(4)° and an average axial Cu–O bond length of 2.35 Å. The bite angle of the ethylenediamine chelate ring, together with other angles about the copper atom, shows a severe distortion of the octahedral geometry. Interestingly, the tetragonal elongation of the axial Cu–O bonds lifts up the N3 atom and pulls down the N4 atom of the ethylenediamine chelate ring, resulting in an envelope conformation of this chelate ring, such that the plane of the N3–N4-containing moiety is severely tilted from that of the N1–N2-containing fragment of the phen ligand, and this results in a significant deviation from the ideal square plane formed by the equatorial donor atoms. As edda moiety has H-donor (N3, N4) and H-acceptor (O2, O4) atoms for H-bonding, the chelate ring conformation of edda may modulate the possible H-bonding interaction of the edda moieties with adjacent base pairs of DNA if the chelated phen moiety intercalates in between the DNA base pairs.

**Figure 13:**
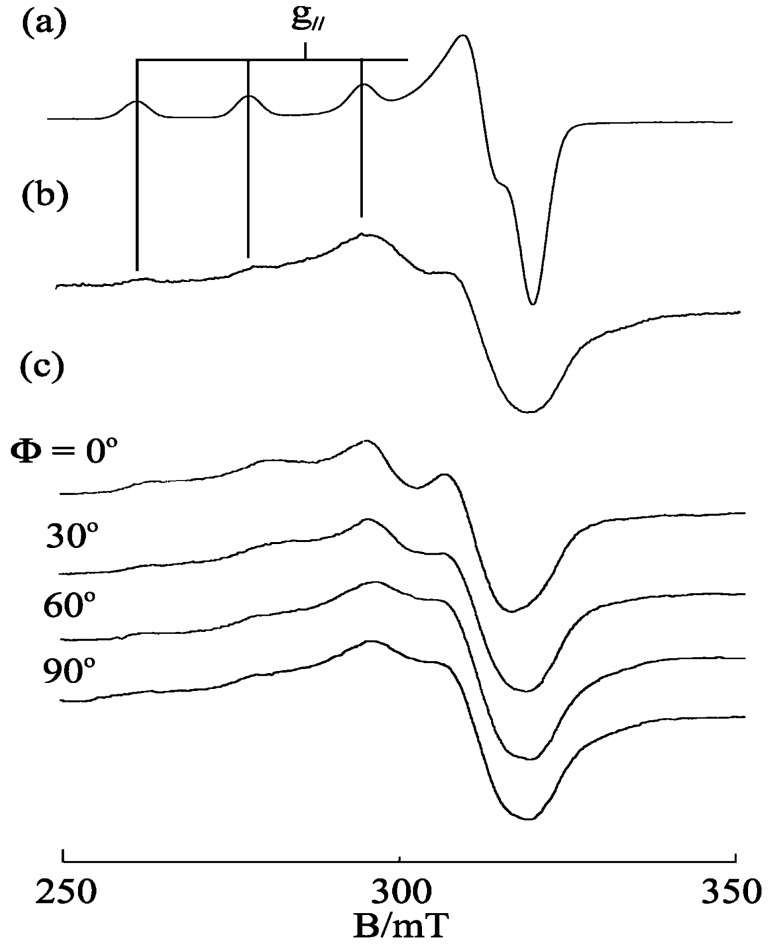
EPR spectra of [Cu(phen)(edda)] (**a**) in frozen solution at −150 °C; (**b**) in DNA-pellet at −150 °C; and (**c**) in A-form DNA fiber at room temperature. Ф is the angle between the fiber axis and the static magnetic field [25].

The EPR spectrum of [Cu(phen)(edda)] in frozen aqueous solution at pH 7 shows a pattern characteristic of a tetragonal Cu(II) complex with *g*_||_ = 2.24 and *A*_||_ = 17.0 mT. However, the EPR spectra of [Cu(phen)(edda)] in DNA pellet and in A-form DNA fiber at room temperature (Figure 13) are broadened considerably, indicating that the copper(II) coordination sphere is deformed inhomogeneously on the DNA. The observed small Ф dependence of the EPR spectra of the A-form DNA fiber indicates that the complex is oriented randomly on the fiber.

**Figure 14:**
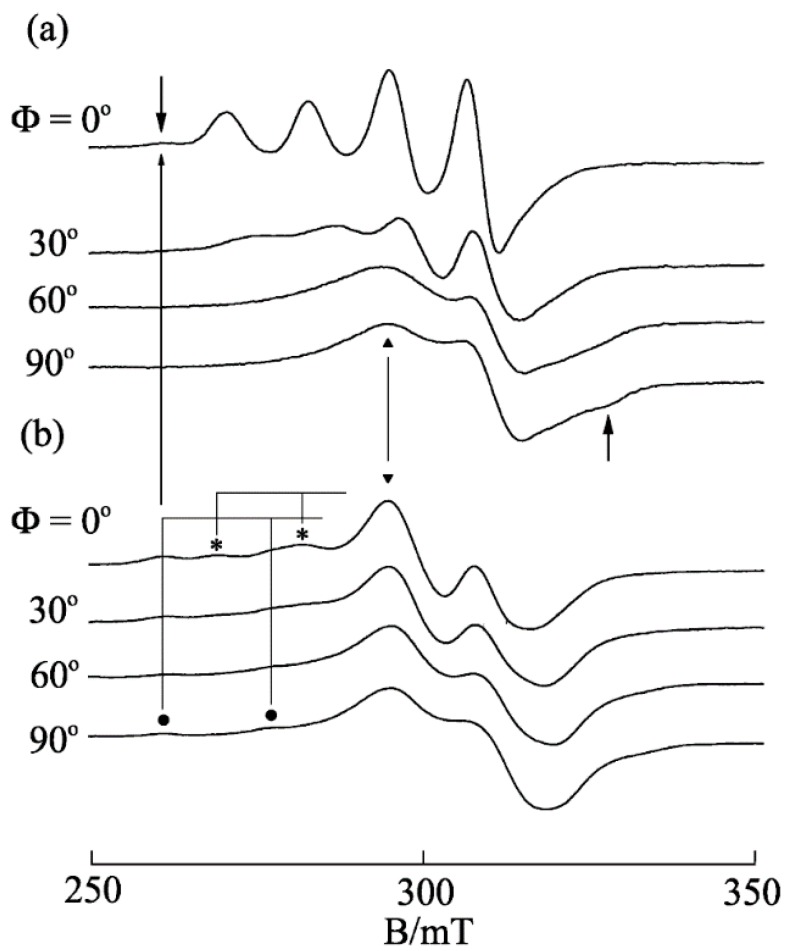
EPR spectra of [Cu(phen)(edda)] in B-form DNA fiber at (**a**) room temperature and (**b**) −150 °C [25].

In contrast to the inhomogeneous broadening of the spectra of [Cu(phen)(edda)] in DNA pellet or in A-form DNA fiber, the EPR line shape changes dramatically with the conformational change in the DNA fiber from A to B (Figure 14). The conspicuous Ф dependence of the EPR spectra of [Cu(phen)(edda)] in B-form DNA fiber at room temperature indicates that [Cu(phen)(edda)] is stereospecifically reoriented with respect to the double-helical DNA axis. The four intense line peaks observed at Ф = 0° indicates that one of the *g* tensor axes (*g*_||_ = 2.20 and *A*_||_ = 12.0 mT) is oriented along the DNA fiber axis. The considerable decrease in the A_||_ value from that observed in frozen solution (*A*_||_ = 17.0 mT) suggests that the tetragonal coordination geometry of [Cu(phen)(edda)] changes considerably in the B-form DNA fiber. It should be noted that additional signals (the arrows in Figure 14a) were observed at the low-field end of the spectrum of Ф = 0° and at the high-field end of the spectrum at Ф = 90°. These signals correspond to another species bound with a different coordination structure.

Freezing of the B-form fiber at −150 °C causes another dramatic change in the EPR line shapes. The four intense line signals observed at Ф = 0° at room temperature are replaced with broad signals over the range 290–330 mT and with several weak peaks in the range 250–285 mT (Figure 14b) [25]. The intense peak that emerges at 295 mT (marked as ▼) indicates that the complex is distorted toward a rhombic symmetry. Though the line shapes observed at low temperature did not change with Ф so conspicuously as those at room temperature, the intensity of the peaks marked with asterisks (*) decreased with increasing Ф, indicating that the species has still some preferred orientation on the DNA fiber. On the other hand, the intensity of the peaks marked with a filled circle (●) does not change with Ф, indicating that the species is randomly oriented on the fiber. The *g*_||_ and *A*_||_ values estimated for latter species are very similar to those estimated for the species in frozen solution and DNA pellet. Interestingly, the magnetic field of the peak observed at the lowest end of the spectra at Ф = 0° in Figure 14a almost coincides with those of the peaks at the lowest end of the spectra of frozen solution and DNA-pellet. Therefore, these signals can be assigned as those of the species bound to the DNA keeping the tetragonal structure observed in the crystal or in frozen solution.

We have shown that the ternary complexes of Cu(II)-phen with various amino acids bind to DNA with different binding modes, *viz*. one intercalative and the other groove bound. In some cases, the amino acid is replaced by the coordinating groups in DNA. However, it has never been observed that the ternary complexes of amino acids are deformed so much toward rhombic form upon binding to DNA. It is evident that the edda moiety in [Cu(phen)(edda)] remains preserved at the copper coordination site on the DNA, because no signals from the dissociated mono-phen Cu(II) species (*g*_||_ = 2.29, *A*_||_ = 0.017 cm^−1^) are discernible in the EPR spectra. These results indicate that [Cu(phen)(edda)] binds to DNA with the phen plane partially intercalated parallel to the DNA base pair plane with the coordination structure of the phen and edda moieties twisted to form rhombic species.

It is obvious that the two axially coordinating carboxyl oxygen atoms of the edda moiety observed in the crystal structure prevent deeper intercalation of the phen moiety and that the interaction of the carboxyl groups with DNA causes in turn the rearrangement of the coordination sphere of the complex on the DNA. It is also possible that the solvated water molecules around the carboxylate groups in [Cu(phen)(edda)] exert stress on the copper coordination sphere when the DNA fibers are frozen to form micro-icebergs in the fibers, resulting in the randomization of the orientation of the copper coordination planes. Though a part of [Cu(phen)(edda)] bound to DNA retains the initial tetragonal structure on the DNA, as judged by the *g*_||_ signals in the EPR spectra of DNA-pellet, one could reasonably presume that the rhombic distortion of [Cu(phen)(edda)] on the DNA is the key to understanding the function of this complex.

### 3.6. Ternary Cu(II) Complexes of Cationic Schiff Bases and N-Heteroaromatic Diimines

**Figure 15:**
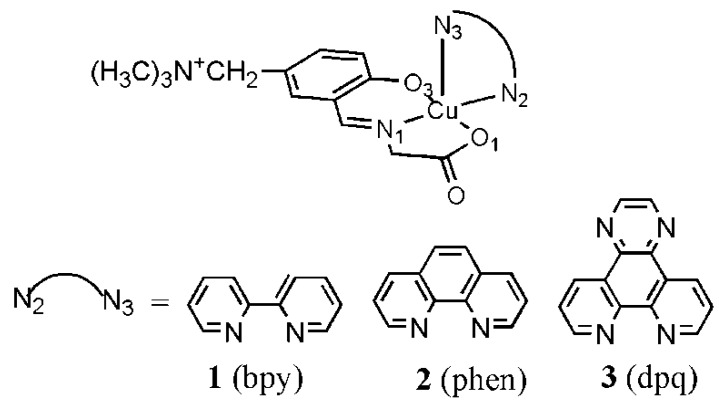
Ternary Cu(II) complexes of cationic Schiff base and diimines. The numbers correspond to the respective Cu(II) complexes [60].

It has been reported that many ternary Cu(II) complexes of Schiff base ligands and diimines show unique properties when they bind and react with DNA [15,16,17,18,19,20,21,22]. With these results in mind, the interaction of ternary Cu(II) complexes of cationic Schiff bases and diimines (Figure 15) with DNA has been investigated by using DNA-fiber EPR, UV-vis, CD, and fluorescence spectroscopy [60]. The oxidative and photodynamic DNA cleavage activities of the complexes have been also discussed in terms of the DNA binding affinities and DNA binding structures.

**Figure 16:**
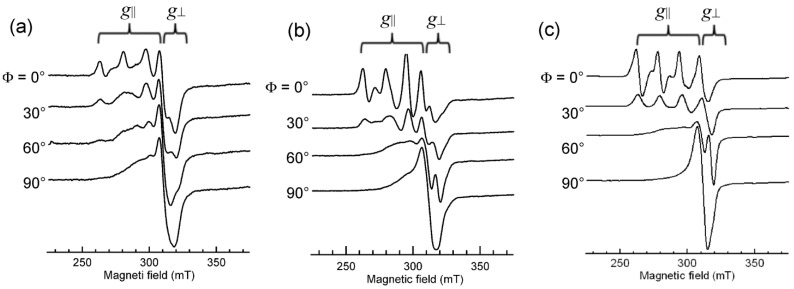
B-form DNA-fiber EPR spectra of (**a**) **1**, (**b**) **2** and (**c**) **3** at room temperature [60]. Φ is the angle between the static magnetic field and the DNA double-helical axis. The DNA fibers were prepared using the buffer solution (20 mM HEPES, 30 mM NaCl (pH 7.4)). [DNA-bp]/[complex] = 20.

The B-form DNA fiber EPR spectra of 1, 2, and 3 are shown in Figure 16. The sharp decrease in intensity of single crystal-like *g*_||_ signals and the increase in intensity of *g*⊥ signals with increase in Φ indicates that the ternary Cu(II) complex with dipyridoquinoxaline (dpq) **3** binds with the *g*_||_ axis parallel to the DNA double-helical axis. As shown in Figure 16a,b, the substitution of bpy (**1**) or phen (**2**) for dpq increases the relative intensity of *g*⊥ signals at Φ = 0°, indicating that the smaller the aromatic amines, the fewer the number of species with the *g*_||_ axis parallel to the DNA double-helical axis. It should also be noted that the magnetic parameters of the complexes in frozen solution changes on binding to the B-form DNA fibers (Table 1). A decrease in the *g*_||_ values and an increase in the *A*_||_ values correspond to the weakening of axial coordination and an increase in the planarity of the complexes, suggesting that the structures of the complexes change upon binding to the DNA [34,35,61]). The X-ray crystal structures of **1** and **2** (Figure S12) indicate that the complexes have tetragonal pyramidal coordination geometries; the basal plane is determined by N1, O1, N2 and O3 atoms and the Cu–N3 bond is much longer than the other coordination bonds, indicating that the *g*_||_ axis, which is parallel to the tetragonal axis, is along the Cu–N3 bond.

**Table 1:** The magnetic parameters.

Complex	Frozen Solution (−150 °C)		B-Form DNA Fiber (r.t.) *	
	*g*_||_	*A*_||_/(10^−4^ cm^−1^)	*g*_||_	*A*_||_/(10^−4^ cm^−1^)
**1**	2.26	165	2.23	184
**2**	2.26	167	2.23	178
**3**	2.26	170	2.23	178

* The values for the intercalated species.

**Figure 17:**
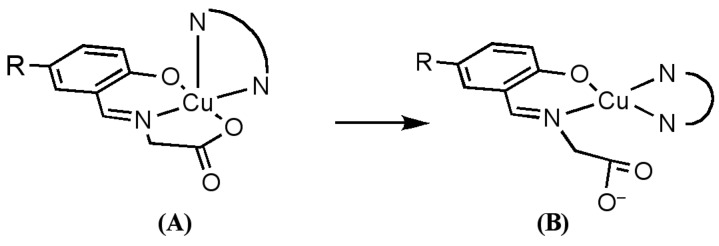
Proposed structural change in ternary Cu(II) complexes of cationic Schiff base and diimines on binding to DNA [60]. (**A**) Structure of the complex in crystal; (**B**) Structure of the complex intercalated to DNA.

If the complexes were intercalated with the *N*-heteroaromatic diimines maintaining the structures in the crystals, the *g*_||_ axes orient perpendicular to the DNA double-helical axis. However, the EPR results indicate that the intercalation of diimines of the ternary Cu(II) complexes of cationic Schiff bases induces structural rearrangement on the Cu(II) coordination spheres, as shown in Figure 17. The *g*_||_ axis of (**B**) is perpendicular to the coordination plane. Thus, the intercalation of the diimines (*N*-heteroaromatic amines) orients the *g*_||_ axis parallel to the DNA double-helical axis.

The DNA binding of the complexes have been also characterized by UV-vis, CD, and fluorescence spectroscopy together with DFT calculation of the structural rearrangement. An analysis of the results revealed that the synergetic effect of intercalation, electrostatic interaction, and rearrangement of the coordination sphere enhances the oxidative and photodynamic DNA cleavage activities of the complexes [60].

### 3.7. Binary Complex of Cu(II) with 2,2′-Bipyridine (bpy)

**Figure 18:**
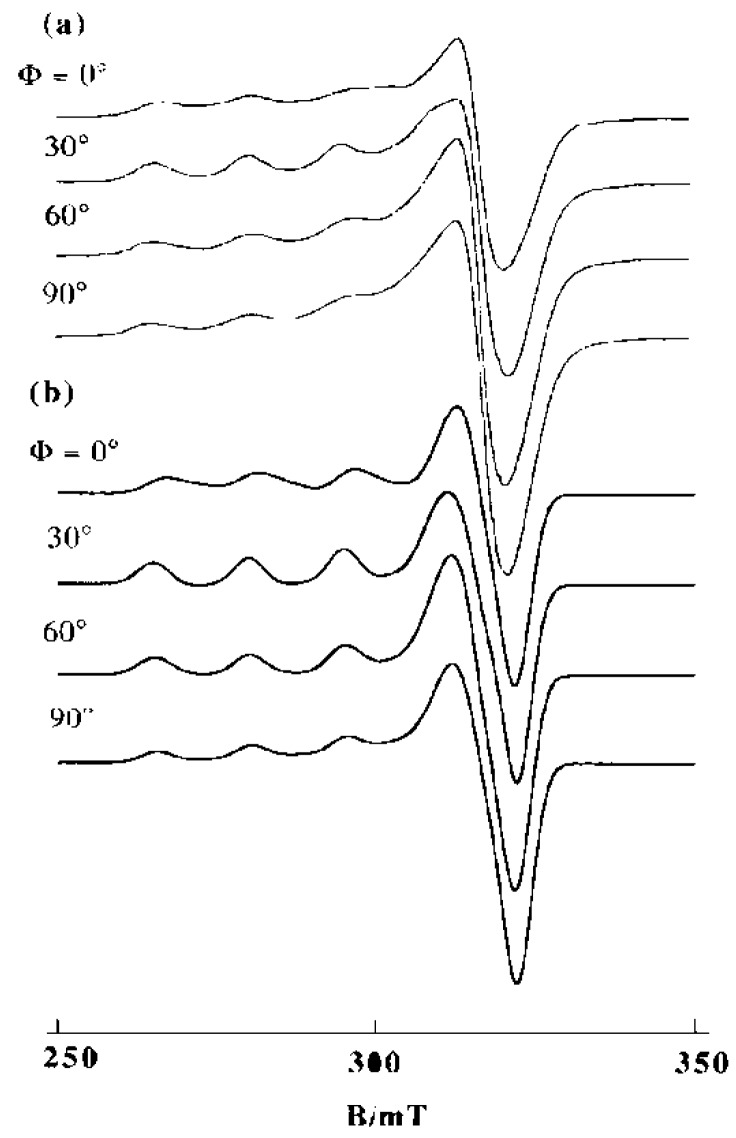
Observed (**a**) and calculated (**b**) EPR spectra of [Cu(bpy)(H_2_O)_3_] on B-form DNA fibers at room temperature. [DNA-bp]/[Cu(II)] = 25, species **A**: θ = 40°, Δθ = 20°; species **B**: randomly oriented. For both **A** and **B**, *g*_||_ = 2.280, *g*⊥  = 2.08, *A*_||_ = 0.0149 cm^−1^, $A_{⊥}$ = 0.0010 cm^−1^*A*_N||_ = 0.0010 cm^−1^, $A_{N⊥}$ = 0.0010 cm^−1^, Δ*B*_||_ = 40 G, Δ$B_{⊥}$ = 40 G [27].

The Ф-dependence of the EPR spectra of mono-bpy Cu(II) complex on A-form DNA fibers is considerably smaller than that of the monophen Cu(II) complex, indicating that the complex is oriented almost randomly on the fibers. On B-form DNA fibers, however, the complex shows a Ф-dependence different from those of both monophen and mono–2,9-dmp Cu(II) complexes (Figure 18a). The *g*_||_ signals become most prominent at Ф = 30°, suggesting that there is a species whose *g*_||_ axis is tilted uniquely relative to the fiber axis. The spectra were simulated by assuming that the two species are on the fiber in the molar ratio 1:0.5. Another assumption is that one of the species is oriented with θ = 40° and Δθ = 20° while the other is oriented randomly. Though the quantitative evaluation of the two species remains rather tentative, it becomes unambiguous that quite a lot of the complex binds to DNA stereospecifically with the *g*_||_ axis tilted by about 40° from the double-helical DNA axis. It should be noted that the magnetic parameters estimated for the complex on B-form DNA fibers at room temperature (*g*_||_ = 2.280, *A*_||_ = 0.0149 cm^−1^) change considerably from those observed (*g*_||_ = 2.307, *A*_||_ = 0.0170 cm^−1^) for the frozen solutions.

It has been reported that the ternary complexes of Pt(II) or Pd(II) with bpy and ethylenediamine intercalate to DNA [62]. Many other investigations have demonstrated the presence of stacking interactions between bpy moiety and the aromatic rings of nucleotides in the ternary complexes of Pt(II) with py and ethylenediamine derivatives [63,64,65,66,67]. Lueth *et al.* reported the importance of intramolecular stacking interactions in the mixed ligand complexes formed by Cu(II), bpy or phen, and 2′-deoxyguanosine-5′-monophosphate [68]. It has also been suggested that the bis-complex [Cu(bpy)2(H_2_O)]^2+^ intercalates or partially intercalates into double-helical DNA [69]. The speculation is based on the observation of an anisotropic EPR spectrum of the complex at room temperature in water and a relatively slow reduction of the complex by ascorbate in the presence of DNA. The present results, however, clearly indicate that about two thirds of the bound mono-bpy Cu(II) species are oriented with the *g*_||_ axis inclined about 40° from the DNA fiber axis, and the others are randomly oriented. The smaller *g*_||_ and *A*_||_ values (*g*_||_ = 2.280, *A*_||_ = 0.015 cm^−1^) estimated for the complex on B-form DNA fibers at room temperature compared to those for the frozen solution (g_||_ = 2.307, *A*_||_ = 0.0170 cm^−1^) and DNA-pellet (*g*_||_ = 2.296, A_||_ = 0.0174 cm^−1^) at −150 °C suggest that the mobility of the complex on the DNA triggers a rearrangement in the Cu(II) coordination structure.

In any event, it is evident that the three fused aromatic rings of phen are critical for the intercalative binding to the double-helical DNA. The observation of the species with θ = 40° for [Cu(bpy)(H_2_O)_3_]^2+^ with a relatively small Δθ value suggests that the complex is bound in a minor groove, as shown in Figure S4b for [Cu(phen)(H_2_O)_3_]^2+^.

## 4. Conclusions

DNA-fiber EPR spectroscopy has been successfully employed to assess the DNA-bound structures of mono-, bis-, and ternary Cu(II) complexes of phen and its methyl derivatives. Even for a simple monophen–Cu(II) complex, both intercalative and non-intercalative binding modes have been detected. The binding of the tris-aqua forms [Cu(phen)(H_2_O)_3_]^2+^, [Cu(5,6-dmp)(H_2_O)_3_]^2+^, and [Cu(2,9-dmp)(H_2_O)_3_]^2+^ to double stranded oligonucleotide 5′-dCGCGAATTCGCG has been studied by using MM/MD/QM calculations. The preliminary results suggest that a stable intercalated structure exists for [Cu(phen)(OH_2_)_3_]^2+^ and the apically coordinated water molecule stays in the groove without any particular interference with the intercalative binding. Similar results have been obtained for [Cu(5,6-dmp)(H_2_O)_3_]^2+^ and [Cu(2,9-dmp)(H_2_O)_3_]^2+^, revealing that the methyl groups on 5,6- and 2,9-positions on the phen ring and the coordinated water molecules do not interfere with the intercalative binding of the complexes. The unusual orientation-dependent EPR spectra observed for the Cu(II)–mono–2,9-dmp complex bound to A- and B-form DNA fibers at low temperature are attributed to the deformation in the coordination structure of the complex from square pyramidal toward trigonal bipyramidal, reorienting the *g*_||_ axis in the 2,9-dmp coordination plane.

The ternary Cu(II) complexes [Cu(phen)(AA)]^2+^ (AA = amino acids) undergo partial dissociation into [Cu(phen)(H_2_O)_3_]^2+^ and the free amino acid on the DNA. The ratio of the amount of the intact ternary complex to the tris-aqua form on DNA depends upon the nature of the amino acid. Thus the ternary complexes of l-lysine, l-arginine, and l-glutamine scarcely dissociate on DNA and show EPR line shapes characteristic of intercalative DNA binding mode. However, the simulated EPR spectra reveal that about two thirds of [Cu(phen)(Lys)]^2+^ are bound to DNA non-intercalatively.

The structures of Cu(II) complexes of 1,10-phenanthroline-derived alkyl amines in aqueous solution around pH 7 change from distorted tetragonal to trigonal bipyramidal when the size of the alkyl group increases. The observed changes in the physicochemical features of the complexes upon binding to DNA suggests that all of the complexes, except the *N-tert*-butylamine derivative, bind to DNA with partial intercalation of the derivatized phen ring in between the DNA base pairs. The highest DNA cleavage activity observed for the copper(II) complex of *N-tert*-butylamine derivative suggests that the bulky *N-tert*-butyl group plays an important role in modifying the coordination environment around Cu(II) upon binding to DNA and hence the Cu(II)/Cu(I) redox potential, leading to the formation of the activated oxidant responsible for the cleavage.

The axially symmetric EPR line shape of [Cu(phen)_2_(H_2_O)]^2+^ in a frozen solution changes to rhombic ones when it binds to DNA. Also, the conformational change in the DNA from A to B and the freezing of the B-form DNA fibers are accompanied by certain structural changes in the complex. The changes in EPR spectra indicate that the [Cu(phen)_2_(H_2_O)_3_]^2+^ complex species has a coordination geometry flexible enough to change between tetragonal pyramidal and trigonal bipyramidal on the DNA. Similar changes in the coordination structure of the ternary complex [Cu(phen)(edda)] are observed on DNA.

The DNA binding mode of Cu(II)-bipy complex has also been examined in comparison with that of its phen analogue and it has been demonstrated that the three fused aromatic rings in 1,10-phenanthroline or its derivatives are essential for the intercalative binding of the phen complexes.

In conclusion, the above results suggest that the flexibility of the Cu(II) coordination sphere is important in conferring efficient oxidative nuclease activities on the complexes, and leads to tuning of the redox potential of the copper site so as to catalytically produce activated oxygen species. Thus the Cu(II)-1,10-phenanthroline complexes are unique systems that should be investigated further for the development of functional metal complexes.

## Supplementary Materials

Supplementary materials can be found at http://www.mdpi.com/1422-0067/16/09/22754/s1.
