# Supplementary material for: Interaction of DNA with Simple and Mixed Ligand Copper(II) Complexes of 1,10-Phenanthrolines as Studied by DNA-Fiber EPR Spectroscopy
Source: Int J Mol Sci. 2015 Sep 21;16(9):22754–80. doi: 10.3390/ijms160922754 (PMC4613334; doi:10.3390/ijms160922754)
Supplement: Supplementary file 1 [file ijms-16-22754-s001.pdf]

Supplementary Information

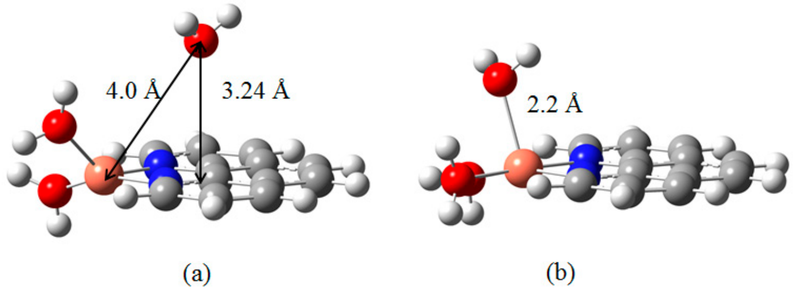

Figure S1. (**a**) Initial geometry of structure-optimized $[Cu(phen)(OH_2)_2]^{2+}$ and a water molecule; (**b**) optimized structure of $[Cu(phen)(OH_2)_3]^{2+}$. The color of the atoms: orange, Cu; blue, N; red, O; gray, C; white, H. The DFT calculations were run on Gaussian 09 Revision D.01 with the M06 functional with the basis sets 6-31G(d,p) for C, N, O, and H and aug-cc-PTZ for Cu(II), taking into account water as a solvent [39]. It was confirmed that the frequency calculation for the optimized structure did not give any imaginary frequency values. The difference in the energy $\Delta E = E(a) - E(b)$ = 0.06504, a.u. = 170.76 kJ/mol.

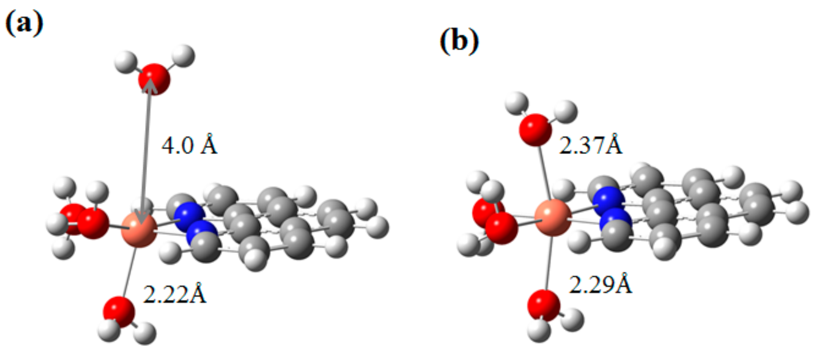

Figure S2. (**a**) Initial geometry of structure-optimized $[Cu(phen)(H_2O)_3]^{2+}$ and a water molecule; (**b**) optimized structure of $[Cu(phen)(H_2O)_4]^{2+}$. The difference in the energy $\Delta E = E(a) - E(b)$ = 0.01832, a.u. = 48.12 kJ/mol. The color of the atoms: orange, Cu; blue, N; red, O; grey, C; white, H.

MM/MD/QM Calculation

The binding structures of the complexes to a double-stranded oligonucleotide 5′-dCGCGAATTCGCG (odn1) were analyzed by molecular mechanics (MM), molecular dynamics (MD), and quantum mechanical (QM) calculations using AMBER14 [40], Gaussian09 [39] and RESP-ESP charge Derive Server [41].

(1). The optimized structures of the complexes were obtained as described in the caption of Figure S1. The Gaussian output files of the complexes were converted to pdb files by GaussView 5.0.

(2). The pdb files were used to evaluate the RESP charge and the force field parameter files (frcmod file) by RESP-ESP charge Derive Server [41]. The force field parameters that could not be determined by the server were estimated tentatively by the trial-and-error method so that the structure optimize MM calculations on AMBER 14 give almost the same structure as the one

obtained by DFT calculations. The "parmcheck" tool on AMBER14 was also used to improve the force field parameters by using the mol2 files made by RESP-ESP charge Derive Server.

(3). The pdb file for odn1 was made by the nucleic acid builder NAB on AMBER14.

(4). The parameter topology file (.prmtop) and input card file (.inpcrd) of the systems were obtained from the odn1 pdb, the complex mol2, and the frcmod files by using xleap tool with leaprc.ff14SB parameter set for odn1 on AMBER 14. The complex was docked manually to odn1, neutralized by adding Na^+, and solvated with 12 Å buffer of TIP4P water octagon.

(5). Minimization and MD calculations were run according to the AMBER basic tutorial B1 (http://ambermd.org/tutorials/basic/tutorial1/). After 1000-step minimization with positional restraints of 500 kcal·mol^{-1}·Å^{-2} for the odn1 and complex, following 2500-step minimization without the restraints, the temperature of the system was gradually raised from 0 to 300 K in 20 ps with weak positional restraints of 10 kcal·mol^{-1}·Å^{-2} at constant volume. Then, MD calculations for more than 20 ns were run at constant pressure (1 atm) and temperature (300 K).

(6). Finally, to refine the binding structures, 10 ps of QM/MD calculations [70] were run, setting the complex as a QM part starting from the restart file obtained after 10–20 ns MM/MD calculations. The DFT calculations for the QM part were made by Gaussian 09 with M06 functional and 6-31G** basis set for the complexes.

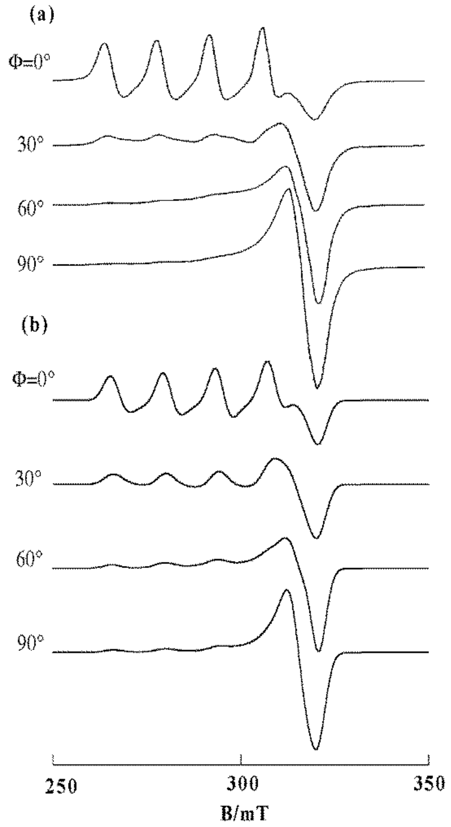

Figure S3. Observed (**a**) and calculated (**b**) EPR spectra of $[Cu(5,6\text{-}dmp)]^{2+}$ on B-form DNA fibers at room temperature. [DNA-bp]/[Cu(II)] = 25, [**G**]/[**I**] = 2. Other parameters used for the simulation; species **I**: θ = 6°, Δθ = 12°, $\Delta B_{\parallel}$ = 40 G, $\Delta B_{\perp}$ = 30 G and species **G**: θ = 30°, Δθ = 30°, $\Delta B_{\parallel}$ = 40 G, $\Delta B_{\perp}$ = 40 G. [**G**]/[**I**] = 1.5. For both **I** and **G**, $g_{\parallel}$ = 2.290, $g_{\perp}$ = 2.08, $A_{\parallel}$ = 0.0154 cm^{-1}, $A_{\perp}$ = 0.0010 cm^{-1}, $A_{N\parallel}$ = 0.0010 cm^{-1}, $A_{N\perp}$ = 0.0010 cm^{-1}.

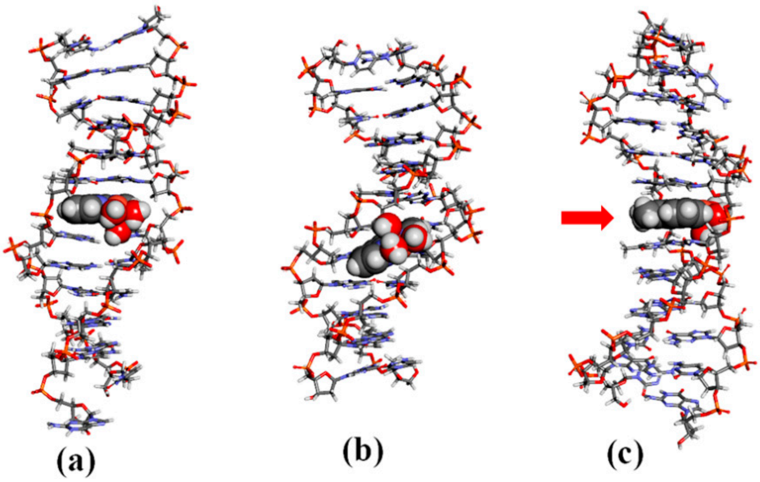

Figure S4. (**a**) Intercalated form of [Cu(phen)(H_2O)$_3$]$^{2+}$; (**b**) minor-groove-bound [Cu(phen)(H_2O)$_3$]$^{2+}$; and (**c**) intercalated form of [Cu(5,6-dmp)(H_2O)$_3$]]$^{2+}$; The red arrow in (**c**) shows the position of 5,6-dimethyl groups. The structures were obtained after 10 ps of QM/MD calculation starting from the structure obtained after 10–20 ns of MM/MD calculations. The color of the atoms in the complex: orange, Cu; blue, N; red, O; grey, C; white, H.

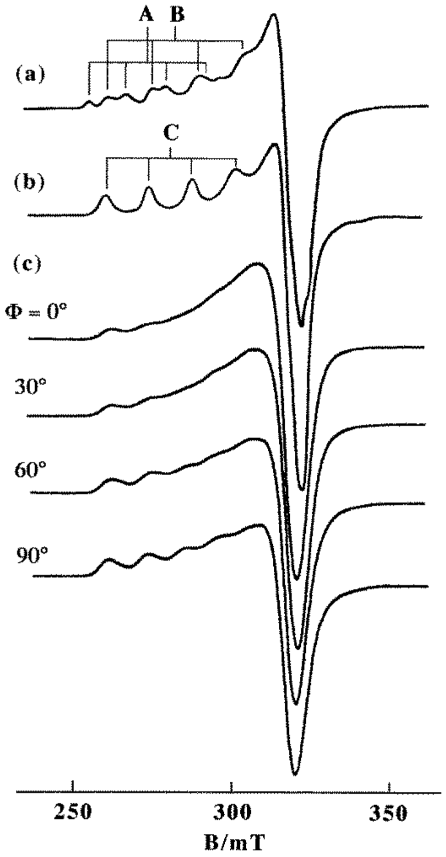

Figure S5. EPR spectra of [Cu(2,9-dmp)(H_2O)$_3$]$^{2+}$ (**a**) in frozen solution at −150 °C, species **A**: $g_{\|}$ = 2.411, $A_{\|}$ = 0.0132 cm^{-1}; species **B**: $g_{\|}$ = 2.332, $A_{\|}$ = 0.0151 cm^{-1}; (**b**) in DNA pellet at −150 °C; species **C**: $g_{\|}$ = 2.346, $A_{\|}$ = 0.0140 cm $^{-1}$; and (**c**) on A-form DNA fibers at room temperature. [DNA-bp]/[Cu(II)] = 30.

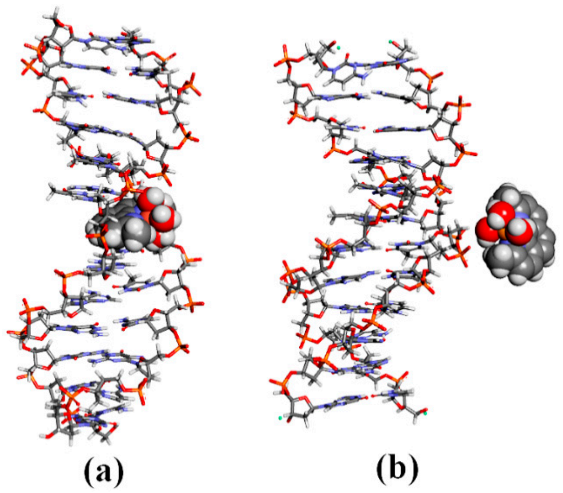

Figure S6. (**a**) Intercalated form of [Cu(2,9-dmp)(H_2O)$_3$]$^{2+}$ after 10 ps of QM/MD calculation starting from the structure obtained after 20 ns of MM/MD calculation; (**b**) snapshot of [Cu(2,9-dmp)(H_2O)$_3$]$^{2+}$ at 25 ns of MM/MD calculation starting from the minor groove bound form. The color of the atoms in the complex: orange, Cu; blue, N; red, O; grey, C; white, H.

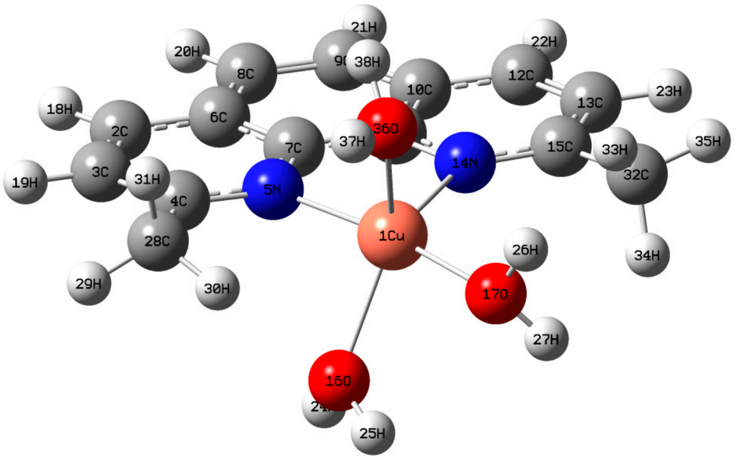

Figure S7. Definition of τ value of [Cu(2,9-dmp)(H_2O)$_3$]$^{2+}$. τ = {angle(17O-1Cu-5N) – angle(16O-1Cu-14N)}/60.

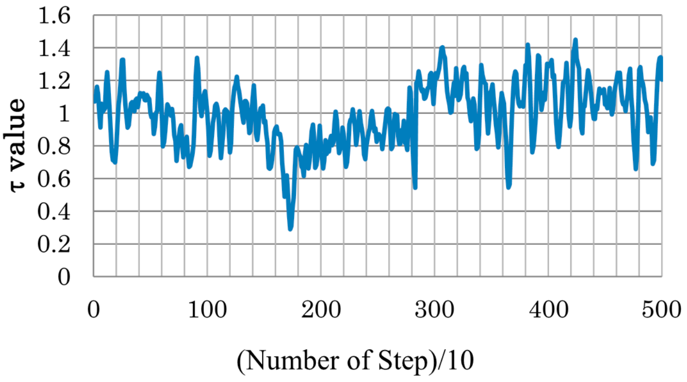

Figure S8. The change in τ value of intercalated [Cu(2,9-dmp)(H_2O)$_3$]$^{2+}$ for 10 ps estimated from the QM/MD calculation (1 step = 0.02 ps).

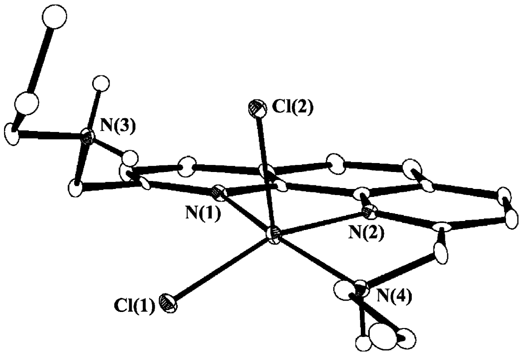

Figure S9. Crystal structure of Cu(II) complex of **2** with atom numbering scheme. The hydrogen atoms have been omitted for clarity [28].

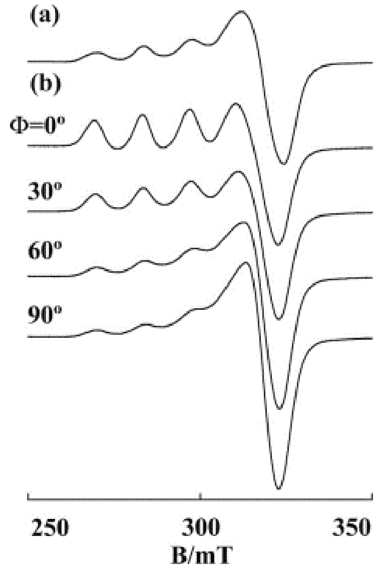

Figure S10. EPR spectra of **1** on DNA pellet at −150 °C (**a**) and on A-form DNA fiber at room temperature (**b**).

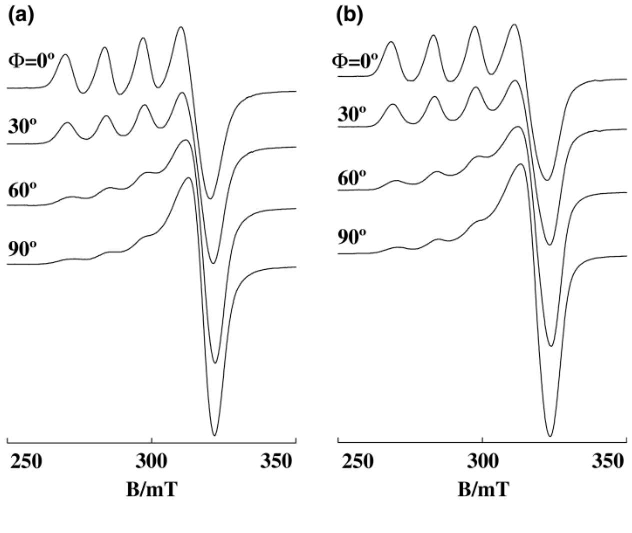

Figure S11. EPR spectra of **1** on B-form DNA fibers at (**a**) room temperature or (**b**) −150 °C.

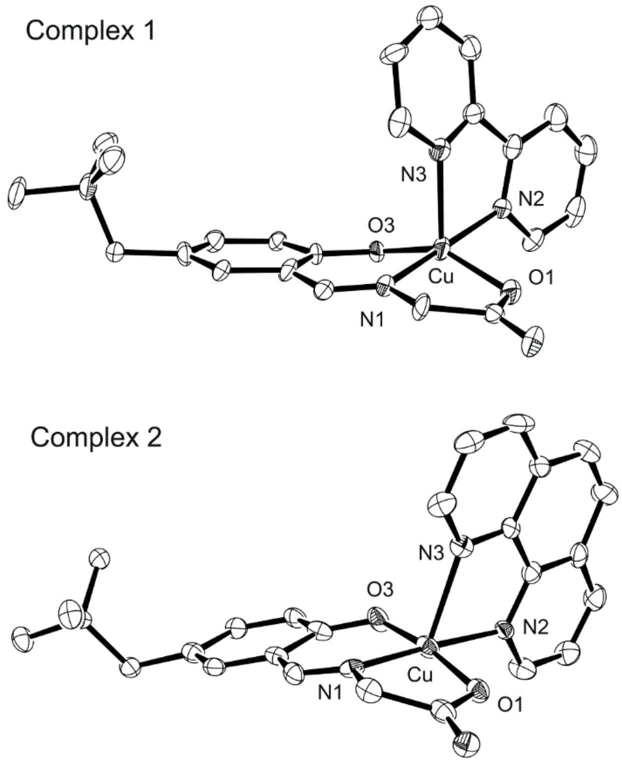

Figure S12. ORTEP view of complexes **1** and **2** [60].
